# Pramipexole Extended Release: A Novel Treatment Option in Parkinson's Disease

**DOI:** 10.4061/2010/612619

**Published:** 2010-12-19

**Authors:** Wolfram Eisenreich, Bernd Sommer, Sebastian Hartter, Wolfgang H. Jost

**Affiliations:** ^1^Boehringer Ingelheim Pharma GmbH & Co. KG, Pharmaceutical Development, 88397 Biberach, Germany; ^2^Boehringer Ingelheim Pharma GmbH & Co. KG, CNS Research, 88397 Biberach, Germany; ^3^Boehringer Ingelheim Pharma GmbH & Co. KG, Drug Metabolism and Pharmacokinetics, 88397 Biberach, Germany; ^4^Department of Neurology, Deutsche Klinik für Diagnostik, Aukammallee 33, 65191 Wiesbaden, Germany

## Abstract

Pramipexole, the most commonly prescribed dopamine agonist worldwide, meanwhile serves as a reference substance for evaluation of new drugs. Based on numerous clinical data and vast experiences, efficacy and safety profiles of this non-ergoline dopamine agonist are well characterized. Since October 2009, an extended-release formulation of pramipexole has been available for symptomatic treatment of Parkinson's disease. Pramipexole administration can be cut down from three times to once a day due to the newly developed extended-release formulation. This is considerable progress in regard to minimizing pill burden and enhancing compliance. Moreover, the 24 h continuous drug release of the once-daily extended-release formulation results in fewer fluctuations in plasma concentrations over time compared to immediate-release pramipexole, given three times daily. The present study summarizes pharmacokinetics and all essential pharmacological and clinical characteristics of the extended-release formulation. In addition, it provides all study data, available so far, with regard to transition and de-novo administration of extended-release formulation for patients with Parkinson's disease. It further compares efficacy and safety data of immediate-release pramipexole with the extended-release formulation of pramipexole.

## 1. Introduction

Parkinson's disease is a chronic neurodegenerative illness that requires regular medication unlimited in time. Parkinsonian patients will comply much more readily to simple therapeutic regimens with fewer applications than to treatments with more frequent drug administration [1]. Pharmacologic developments of improved dopaminergic medication—which has been established as an efficient way of controlling motor symptoms in idiopathic Parkinson's syndrome—accordingly focus on the reduction of the tablet load and on simple treatment options that are convenient for the patient. The objective is to enhance better compliance and consecutively optimum long-term outcomes by creating a more acceptable form of application—that is, an extended-release preparation that has to be taken only once a day. 

A prolonged-action drug permits the steady release of the active ingredient over 24 hours preventing the active concentration from continuously surging on and surging off as commonly encountered with multiple applications of the substance. An unvarying, efficient level by continuous release over 24 hours will help avoid losses during the course of the day. This is nurtured by the hope for better control of motor symptoms during the day and for less nocturnal and matutinal complaints, akinesia for instance, aside from late motor complications in the long run. At the same time, undesired effects might be abated since peak concentrations no longer occur. 

Pramipexole, the dopamine agonist that had been a non-depot formula so far, which patients had to take tid (3x) daily, could be turned into a controlled-release drug to be administered once daily. This paper gives an overview regarding the development and pharmacology of the extended-release form of pramipexole, and it summarizes the available clinical data on depot pramipexole in the treatment of early and advanced Parkinson's disease.

## 2. Pharmacology and Mechanism of Action

Pramipexole (brand name MIRAPEX, SIFROL) is a selective dopamine D2 receptor agonist, approved since 1998. Pramipexole is indicated for the symptomatic treatment of Parkinson's disease (idiopathic Parkinson's syndrome), either alone (without levodopa) or in combination with levodopa, that is, during the entire progress of disease up to the advanced stage. 

Pramipexole is a full nonergoline dopamine agonist with selective affinity for dopamine receptors of the D_2_ subfamily. This substance shows a 7- to 10-fold higher affinity to D_3_ (Ki = 0.5 nM) than to D_2_ receptors (Ki = 3.9 nM). Pramipexole acts on presynaptic as well as on postsynaptic receptors [2, 3]. In intact dopaminergic systems, though, the effects of pramipexole are primarily stirred via presynaptic autoreceptors of the D_3_ and D_2_ type, thereby reducing the synthesis and synaptic release of dopamine. Effects on postsynaptic receptors are only observed at higher doses, and they are marked by prolonged latency periods. With reduced dopamine release due to loss or damage of the presynaptic termini, however, the postsynaptic D_2_ and D_3_ receptors are additionally and immediately stimulated. 

Pramipexole mediates its therapeutic action on motor deficits in Parkinson's disease via the postsynaptic D_2_ and D_3_ receptors of the direct and indirect striatofugal nerve tracts. Here, pramipexole corrects the increased inhibitory impact of the direct pathway on motor activity by enhancing the direct transmission (through D_3_ receptors) and reducing the indirect transmission (through D_2_ receptors) [4].

## 3. Objectives in Developing the Controlled-Release Form

Until 2009, immediate release pramipexole tablets had been offered at a dosage of 0.125 mg, 0.25 mg, 0.5 mg, and 1.0 mg salt (equivalent to 0.18, 0.35, and 0.7 mg base). Owing to their half-life of 8–12 hours, these immediate-release pramipexole tablets are taken tid in 3 equal doses to ensure the daily requirement. It has been an ardent desire of patients, doctors, care persons/family members to reduce the number of applications to a once-daily regimen. Other goals in the development of a controlled-release pramipexole formula were the simple 1 : 1 conversion from immediate-release pramipexole to extended-release pramipexole tablets overnight, a similar or improved peak/trough fluctuation, comparable plasma concentrations and comparable efficacy between the two formulas. Another significant objective was preventing the sudden and uncontrolled release of the active agent by all means (= “dose dumping”) under fasting conditions, after the ingestion of food and also of alcohol, which could lead to increased adverse effects. 

The drug substance pramipexole possesses numerous properties that encumbered development of a prolonged-release formula [5]. Due to its excellent efficacy, pramipexole is very low dosed, which demands a particular standard for the accuracy of dosage. Pramipexole is, moreover, well soluble in water and physiologic fluids such as gastric and enteric juice. This is ideal grounds for immediate-release pramipexole formulas but a great challenge in terms of deliberately delayed release. Immediate-release pramipexole tablets are offered in many potencies to titrate the patients to the appropriate right dose. One therefore tried to develop a great number of depot pramipexole dosages with an identical release of the active agent and dose-linear bioavailability.

## 4. Galenics and Absorption Profile

**Figure 1:**
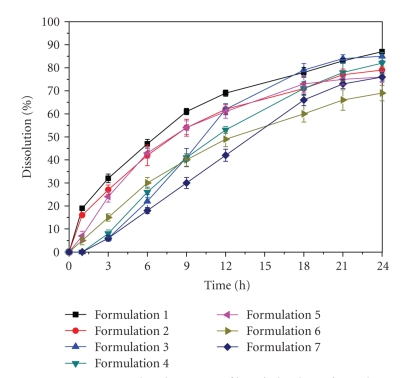
In vitro dissolution profile of the best formulation prototypes (Boehringer Ingelheim).

Several hundreds of pharmaceutical formulas were developed in view of these difficult challenges. There are so-called in vitro dissolution tests to test the behavior of prototypes. As depicted in Figure 1, seven formulas five pellet formulas and two so-called matrix tablets among them presented with a highly promising in vitro release profile. 

All of these seven prototypes were also tested in a clinical phase I study. In a crossover study with in healthy male subjects [6], the pharmacokinetic properties were investigated in the steady state. An optimal formula for further clinical development was supposed to be identified based on the following target criteria:

1. a maximum plasma concentration (C_max,ss_), in the steady state (day 4 of each extended-release pramipexole therapy), that does not exceed the one obtained after immediate-release pramipexole t.i.d., and a minimum plasma concentration (C_min,ss_) not less than the one observed after the immediate-release formula applied t.i.d,
2. a peak/trough fluctuation (PTF) over 24 hours that is smaller than or comparable to the one following immediate-release pramipexole,
3. a bioavailability not less than ≥75% in the steady state that compares with the immediate-release formula,
4. no incidence of irregular release (“dose-dumping” defined as a not more than 80% dose absorption within 4 hours after application).

One matrix tablet (formula 2, Figure 1) met all of the above criteria and was chosen for further clinical development. It consists of an innovative hydrogel formula.With this kind of tablet, the drug substance is homogeneously embedded in a polymeric matrix. The slow down (= extended release) of the agent is accomplished by combining three polymers hypromellose, corn starch, and carbomer. The interaction of these three polymers warrants the uniform and long-lasting release of the agent over 24 hours. Upon contact with digestive juices, the drug substance is first dissolved at the surface. The matrix then starts swelling, forming a viscous jelly that releases pramipexole consistently over 24 hours. Because of pramipexole's good solubility independent from pH-value, the drug substance is dissolved from the matrix also in deeper intestinal segments and is ready for absorption. We do not found a relevant influence on gastric emptying or intestinal motility. 

In vitro tests showed that the consumption of alcohol does not impair the matrix structure. A sudden “dose dumping” can thus be excluded.

## 5. Pharmacokinetics

A level A in vitro/in vivo correlation (IVIVC) with the chosen formula was established in the course that was able to predict a complete plasma concentration profile in humans by in vitro solubility charts [7] with adequate precision. This IVIVC also showed that pramipexole of the extended-release formula was steadily absorbed all through the entire intestine including the colon. The variability among other individuals in this process was rather low and not influenced by other factors food for instance (Table 1). 

In a relative study on bioavailability, pharmacokinetic parameters were compared after the highest daily dose of 3.15 mg (base, equivalent to 4.5 mg salt), administered as extended-release pramipexole once daily (q.d.) or as immediate-release pramipexole, 3x daily (t.i.d.) 1.05 mg base. The impact of food on the pharmacokinetics of extended-release pramipexole was additionally studied. The results were indicative of equivalent pramipexole plasma concentrations in the time span defined as surface on the chart (AUC_0–24,ss_). There were no further relevant differences in the maximum (C_max,ss_) and minimum concentrations (C_pre,ss_-measured right before the first morning dose). No significant differences versus food ingestion on an empty stomach [8] were seen when long-acting pramipexole was taken immediately after a hypercaloric meal. 

**Figure 2:**
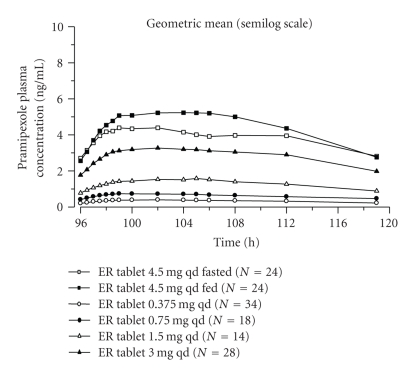
Geometric mean of the pramipexole plasma-concentration time profile at steady state after intake of pramipexole ER (dose range: 0.375 mg to 3.0 mg/d) [6].

This study furthermore corroborated that pramipexole plasma concentrations in healthy male subjects increased in proportion to the doses of extended-release pramipexole, as demonstrated in Figure 2. As this also holds true for the immediate-release formula, patients can be switched to the prolonged action formula for the night, whilst the daily dose is maintained [6].

**Figure 3:**
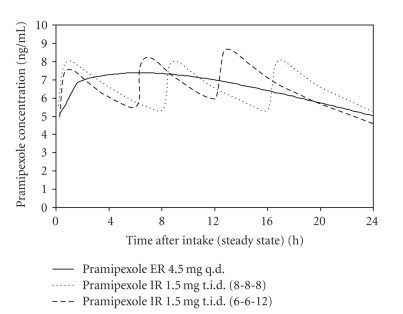
simulated concentration time profile of pramipexole ER and pramipexole IR at steady state in Parkinson patients after intake of pramipexole ER 4.5 mg q.d. or pramipexole t.i.d.) in intervals of 8 hours (8-8-8) or different intervals of 6 and 12 hours (6-6-12) [9].

Based on the results of this study on relative bioavailability, major approval studies used extended-release pramipexole and immediate-release pramipexole in identical daily doses, titration steps, and titration intervals. In a phase III study on patients with early Parkinson's disease, plasma concentrations were determined before taking the morning dose, and 1, 2, and 4 hours later after a stable final dose of pramipexole had been established. Aided by population-centered pharmacokinetic procedures, this enabled the simulation of mean consecutive time profiles of pramipexole plasma concentrations in Parkinson patients as seen in Figure 3 [9]. It illustrates the desired 24 hours steady exposure in patients after the application of the extended-release pramipexole tablet. 

All clinical studies confirmed that matrix tablets are best suited to make for the extended-release of pramipexole [10]. All developmental goals were attained, and, since October 2009, this new product has been approved in many European countries and the U.S. 

In addition to the reliable immediate-release pramipexole tablets, we can avail ourselves now of extended-release pramipexole tablets. These are applied only once a day to reach the daily requirement. Seven dosages have been approved for the time being: 

0.375, 0.75, 1. 5, 2.25, 3, 3.75, and 4.5 mg (S)-2-amino-4,5,6,7-tetrahydro-6-propylamino-benzothiazole dihydrochloride monohydrate equivalent to 0.26, 0.52, 1.05, 1.57, 2.1, 2.62, or 3.15 mg pramipexole base.

## 6. Clinical Data

Pramipexole is the most commonly prescribed dopamine agonist worldwide and may well be considered the reference substance in the evaluation of new agents. Many controlled studies have been published; the efficacy and the efficiency activity are well documented [11]. Extended-release pramipexole (depot) was accordingly compared in studies with immediate-release pramipexole. From clinical viewpoints, the following aspects were focused on before the introduction of the prolonged-action preparation:

- change from immediate-release pramipexole to depot pramipexole,
- effectiveness of depot pramipexole in the early stage of disease,
- effectiveness of depot pramipexole in advanced Parkinson's disease.

## 7. Switching from Immediate-Release Pramipexole to Extended-Release Pramipexole

**Figure 4:**
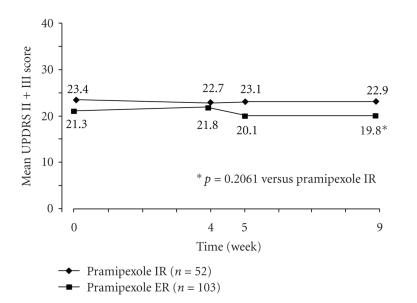
Efficacy of pramipexole IR versus pramipexole ER before and after switch (week 4) comparing the change of mean UPDRS score II + III [12].

Switching a patient from immediate-release pramipexole to extended-release pramipexole is preferably done overnight. Rascol et al. [12] investigated to what extent that could be done. In a double-blind study, 156 patients were switched who had received invariably more than 1.05 mg (base) pramipexole for at least 4 weeks (on the average 1.9 mg (base) immediate-release pramipexole). Changes up to 15% in the UPDRS **(**Unified Parkinson Disease Rating Scale) II and III were rated noninferior. 84.5% were successfully changed overnight (87 out of 103 patients). The proof on noninferiority failed although extended-release pramipexole turned out even better per UPDRS and CGI-I (Clinical Global Impression Improvement), responder rate (87. 4 versus 78.8%) (Figure 4). There was no difference in side effects. 

**Figure 5:**
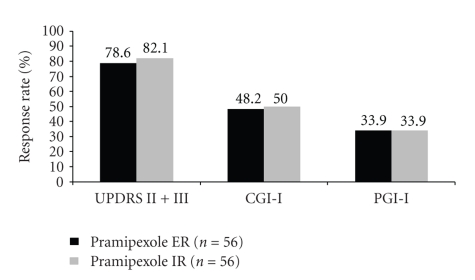
Response rates of pramipexole IR versus pramipexole ER. Comparison of the efficacy parameters UPDRS II + II, CGI-I and PGI at study endpoint (week 12, FAS) [13].

A second switch study was conducted by Mizuno et al. in Japan [13], also making use of the overnight approach. This double-blind study was run for 12 weeks in 112 patients, followed by a 4-week open phase. The patients had a mean 3 years' history of PD (2.9 and/or 3.1). The efficacy was assessed by UPDRS part II and III. Differences in UPDRS-rating were irrelevant (immediate-release pramipexole 13.3, extended-release pramipexole 13.6): this also pertained to the data of CGI-I and PGI-I (see Figures 4 and 5). The switch was successful in 83%. The rate of adverse effects did not differ. 

In sum, the switch was uneventful in approximately 85% of the patients. The remainder did not complain about major side effects; they even indicated a better response under immediate-release pramipexole.

## 8. Effectiveness of Extended-Release Pramipexole in the Early Stage of Disease

The most valuable study to address this question was conducted by Poewe et al. [14]. This work group scrutinized 539 patients who had had symptoms for averagely 12 months and treated them with immediate-release pramipexole by 3-arm design: depot pramipexole: placebo in a ratio of 2 : 2 : 1 for 26 weeks. The effectiveness was checked upon after 18 weeks, the (noninferiority) after 33 weeks. UPDRS II and III besides CGI-I (Clinical Global Impression Improvement) and PGI-I (Patient Global Impression Improvement) served as parameters. The two goals of the study were attained, that is, proof of the superiority versus placebo in week 18 and proof of the non-inferiority of extended-release pramipexole in week 33 (−8.6 versus −8.8 scores). Relevant differences in respect of CGI-I and PGI-I were not seen (46.1/33.3 versus 43.3/34.4%), The incidence and severity of side effects did not differ either. 

Another important interim analysis in this type of patients is owed to Salin et al. [15]. In this 3-arm study, 101 patients were examined for 33 weeks. 84 patients were evaluated at the end of the study, 35 of them being on extended-release pramipexole, 31 under immediate-release pramipexole and 18 under placebo. Changes of UPDRS II and III were compared with week 18 to 33. The group taking extended-release pramipexole scored higher by a change of 0.3 (−11.8 versus −11.5 points). The group treated with immediate-release pramipexole presented with a change of −11.9 points. The change in the placebo group amounted to −4.2 points up to week 18 and −2.7 points to week 33. This means that in both drug groups the effect was maintained between week 18 and 33 whereas a decline of 1.5 points in was recorded in the placebo group. 

Parallel data on response were also collected. PGI interestingly enough revealed a slight superiority of the extended-release formula compared to immediate-release pramipexole in week 18 and 33 (45.7/42.9 versus 35.5/41.9%), evaluation of CGI disclosed a superiority of the immediate-release formula in week 18 and 33 (64.5/51.6 versus 46.9/40.6%).

Another analysis on extended-release pramipexole in the early stage of PD was published by Hauser et al. [16]. Enrolled were 259 patients who had been ill for about a year. The study was carried on for 18 weeks with an initial titration period of 7 weeks). This controlled double-blind trial (extended-release pramipexole: immediate-release pramipexole: placebo = 2 : 2 : 1) also focused on the effect by employing UPDRS II and III plus CGI-I and PGI-I. An improvement by 7.5 points was found in the group treated with immediate-release pramipexole (*n* = 99): the group taking extended-release pramipexole (*n* = 99) scored 7.4 points and the placebo group (*n* = 42) 2.7 points. Response rates were determined by GCI-I and PGI-I and were indicative of improvements in week 18 and 33 for extended-release pramipexole in 37/35.6%. The result was 48/23.8% under immediate-release pramipexole and 18/12% under placebo.

The authors concluded that both pramipexole formulas were safe and superior to placebo, aside from being well tolerated. The efficacy of extended-release pramipexole was said to be similar to immediate-release pramipexole with comparable adverse effects.

## 9. Effectiveness of Extended-Release Pramipexole in Advanced Parkinson's Disease

**Figure 6:**
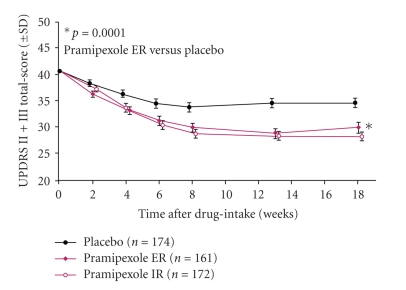
Efficacy of Pramipexole ER in patients with advanced Parkinson's disease compared to pramipexole IR and placebo [17].

The most significant study on extended-release pramipexole in the advanced stage of Parkinson's disease was carried out by Schapira et al. [17]. This examination included 517 patients, who had been diseased for about 6 years, treated already by approximately 600 mg levodopa. Used in a 3-arm design were immediate-release pramipexole: extended-release pramipexol: placebo in a ratio of 1 : 1 : 1. The superiority of pramipexole was studied after 18 weeks by UPDRS part II and III (Figure 6) and by off periods. The effectiveness was rechecked then 33 weeks later (*n* = 385). Extended-release pramipexole proved superior after 18 weeks, reflected by 4.9 points (−11.0 versus 6.1) in UPDRS and 0.7 (−2.1 versus 1.4) hours off time as opposed to placebo. There were no relevant differences between immediate-release pramipexole and extended-release pramipexole. The great effect of placebo was astonishing. Giddiness and vomiting occurred less under extended-relapse pramipexole than under immediate-release pramipexole.

## 10. Consequences in Clinical Practice

The aforementioned studies confirm preclinical data. Immediate-release pramipexole and extended-release pramipexole only vary in releasing the active agent from the tablet. The substance itself is unchanged, meaning there is an identical receptor profile, identical efficacy, and identical receptor binding. The half-life of the agent is also the same, but the continuous release from the depot tablet results in an overall prolonged plasma half life. Any of the accepted statements linked to immediate-release pramipexole also apply to extended-release pramipexole-except for the daily doses required. Extended-release pramipexole can be used in both early and late Parkinson's disease. A switch can take place overnight, the ratio being 1:1, for example, immediate-release pramipexole 3 × 0.7 mg (base) are consistent with 2.1 mg extended-release pramipexole. In most cases, the patient is not going to be affected by this switch. More efficaciousness, increased dyskinesias or undesired effects are not to be expected. In a few cases, the effect may be less, which would require a dose adjustment. Some patients appreciate the stimulating effect of immediate-release pramipexole with a rapid afflux and higher peak and for this reason, they prefer fast-release pramipexole. So far, there have been no insights regarding any increased individual side effect owing to the use of extended-release pramipexole. Reduction of plasma peaks might even result in decreased side effects. 

The once daily application constitutes the essential and cardinal advantage of the extended-release formula. This is definitely going to improve compliance, and to ameliorate disease control. The argument that the patient gets levodopa 3 times a day anyways does not hold, since levodopa is taken food dependent (competitive action of alimentary amino acids to levodopa during its resorption [18]); a 3x daily scheme can well be turned into a 6x daily one. Extended-release pramipexole can also be split into 2 administrations if clinically applicable albeit this is not provided for in the approval. 

Since the introduction of extended-release pramipexole, and in consideration of pharmacologic and clinical aspects, it has been recommended putting the patient on extended-release pramipexole in a new approach. All patients who have been under immediate-release pramipexole may furthermore be switched to extended-release pramipexole overnight. The inauguration of extended release-pramipexole is to be regarded as another important option in the drug treatment of Parkinson's disease.

## Figures and Tables

**Table 1:** Comparison of pharmacokinetic parameters in steady state after intake of Pramipexole IR 1.5 mg t.i.d., Pramipexole ER 4.5 mg q.d. fasted, and Pramipexole ER 4.5 mg q.d. after fat and protein-rich meal.

	1.5 mg Pramipexole IR tablet t.i.d. (*n* = 24)		4.5 mg Pramipexole ER tablet q.d. fasted (*n* = 24)		4.5 mg Pramipexole ER tablet q.d. fed (*n* = 24)	
	gMean	gCV (%)	gMean	gCV (%)	gMean	gCV (%)
AUC0-24,ss (ng·h/ml)	94.4	21.4	91.7	30.1	105	29.6
Cmax,ss (ng/ml)	5.3	19.0	4.9	22.3	5.9	24.7
Cpre,ss (ng/ml)	2.8	27.7	2.7	31.1	2.6	58.4

gMean: geometric Mean; gCV: geometric variation coefficient.

## References

[1] Grosset KA, Bone I, Grosset DG (2005). Suboptimal medication adherence in Parkinson’s disease. *Movement Disorders*.

[2] Dziedzicka-Wasylewska M, Ferrari F, Johnson RD (2001). Mechanisms of action of pramipexole: effects on receptors. *Reviews in Contemporary Pharmacotherapy*.

[3] Kvernmo T, Härtter S, Burger E (2006). A review of the receptor-binding and pharmacokinetic properties of dopamine agonists. *Clinical Therapeutics*.

[4] Mierau J, Schneider FJ, Ensinger HA, Chio CL, Lajiness ME, Huff RM (1995). Pramipexole binding and activation of cloned and expressed dopamine D, D and D receptors. *European Journal of Pharmacology*.

[5] Friedl T, Härtter S, Eisenreich WJ Development of a once daily formulation for Pramipexole.

[6] Jenner P, Könen-Bergmann M, Schepers C, Haertter S (2009). Pharmacokinetics of a Once-Daily extended-release formulation of pramipexole in healthy male volunteers: three studies. *Clinical Therapeutics*.

[7] Koenen-Bergmann M, Haertter S, Schepers C A multiple rising-dose bioequivalence phase I study with a pramipexole extended release (ER) formulation.

[8] European Medicines Agency Pramipexole (Mirapexin® prolonged-release tablets): summary of product characteristics. http//www.emea.europa.eu/humandocs/PDFs/EPAR/Sifrol/emea-combined-h133en.pdf.

[9] Dansirikul C, Staab A, Salin L, Haertter S, Lehr T Population pharmacokinetic analysis of pramipexole extended-release formulation in Parkinson’s disease (PD) patients.

[10] Chwieduk CM, Curran MP (2010). Pramipexole extended release: in parkinsons disease. *CNS Drugs*.

[11] Möller JC, Oertel WH (2005). Pramipexole in the treatment of Parkinson’s disease: new developments. *Expert Review of Neurotherapeutics*.

[12] Rascol O, Barone P, Debieuvre CD (2009). Overnight switching from immediate- to extended-release (ER) in early Parkinson’s disease. *Neurology*.

[13] Mizuno Y, Yamamoto M, Kuno S, Hasegawa K, Hattori N, Kagimura T Efficacy of pramipexole extended release (ER) and switching from pramipexole immediate release (IR) to ER in Japanese advanced Parkinson’s disease (PD) patients.

[14] Poewe W, Barone P, Hauser RA (2009). Pramipexole extended-release is effective in early Parkinson’s disease. *Movement Disorders*.

[15] Salin L, Hauser R, Koester  J Double-blind evaluation of maintenance of efficacy of pramipexole extended-release in early Parkinson’s disease.

[16] Hauser R, Salin L, Koester J (2009). Double-blind evaluation of pramipexole extended-release (ER) in early Parkinson’s disease. *Neurology*.

[17] Schapira A, Barone P, Hauser RA Efficacy and safety of pramipexole extended-release for advanced Parkinson’s disease.

[18] Jost WH (2010). Gastrointestinal dysfunction in Parkinson’s Disease. *Journal of the Neurological Sciences*.

